# Compositional dependency on dissolution rate and cytocompatibility of phosphate-based glasses: Effect of B_2_O_3_ and Fe_2_O_3_ addition

**DOI:** 10.1177/2041731417744454

**Published:** 2017-12-11

**Authors:** Nusrat Sharmin, Fu Gu, Ifty Ahmed, Andrew J Parsons

**Affiliations:** 1 Department of Chemical and Environmental Engineering, Faculty of Science and Engineering, University of Nottingham Ningbo China, Ningbo, China; 2 Ningbo Nottingham International Academy for Marine Economy and Technology, University of Nottingham Ningbo China, Ningbo, China; 3 Ningbo Nottingham New Materials Institute, University of Nottingham Ningbo China, Ningbo, China; 4 Advanced Materials Research Group, Healthcare Technologies, Faculty of Engineering, University of Nottingham, Nottingham, UK; 5 Composites Research Group, Healthcare Technologies, Faculty of Engineering, University of Nottingham, Nottingham, UK

**Keywords:** Phosphate-based glasses, dissolution properties, thermal properties, fibre dissolution mode, cytocompatibility

## Abstract

The unique property of phosphate-based glasses and fibres to be completely dissolved in aqueous media is largely dependent on the glass composition. This article focuses on investigating the effect of replacing Na_2_O with 3 and 5 mol% Fe_2_O_3_ on cytocompatibility, thermal and dissolution properties of P_2_O_5_–CaO–Na_2_O–MgO–B_2_O_3_ glass system, where P_2_O_5_ content was fixed at 45 mol%. The effect of increasing Fe_2_O_3_ from 3 to 5 mol% on P_2_O_5_–CaO–Na_2_O–MgO glasses was also evaluated. The glass transition temperature, onset of crystallisation temperature and liquidus temperature were found to decrease with increasing Fe_2_O_3_ content and the addition of B_2_O_3_, while the thermal expansion values were found to decrease. The density of the glasses decreased with increasing Fe_2_O_3_ content. However, an increase in the density was observed by the addition of 5 mol% B_2_O_3_. The dissolution properties and mode of bulk glass and fibres were also examined which were found to decrease with increasing B_2_O_3_ and Fe_2_O_3_. However, it was found that the dissolution properties of the glasses containing both B_2_O_3_ and Fe_2_O_3_ were lower than only Fe_2_O_3_ containing glasses. The in vitro cell culture studies using human osteoblast like (MG63) cell lines revealed that the glasses containing both B_2_O_3_ and Fe_2_O_3_ maintained and showed higher cell viability as compared to the only Fe_2_O_3_ containing glasses. Glasses containing both B_2_O_3_ and Fe_2_O_3_ showed a pronounced effect on the dissolution rate of the glasses, which eventually improved the cytocompatibility properties of the glasses investigated.

## Introduction

The properties of phosphate-based glasses (PBGs) such as glass transition temperature, thermal expansion coefficient, density, molar volume and dissolution rate strongly depend on the amount and type of modifying oxides added to the glass structure.^1^ Addition of metal cations with higher valency can potentially increase the cross-linking with the glass structure which can decrease the dissolution rate of the glass.^2^ Moreover, addition of cations with higher field strength can also decrease the dissolution rate via increasing the covalent character of the bonds within the glass structure.^3^ The dissolution rate of PBGs is also dependent on P_2_O_5_ content, although the effect is less significant as compared to that of metal cations.^4^ The ability of PBGs to tune the dissolution rate has prompted interest in using these glasses for different biomedical applications.^5,6^ One more unique property of PBGs is the ability of these glasses to be converted into fibres which could be used as a reinforcement of different bioresorbable polymers to produce totally bioresorbable composites for use in fracture fixation devices.^7–10^

A number of glass systems have been developed by addition of various metal oxides such as Fe_2_O_3_, Al_2_O_3_, ZnO, TiO_2_, B_2_O_3_ and SrO for hard tissue engineering applications. Effect of Fe_2_O_3_, CaO and MgO addition on the dissolution property of P_2_O_5_–Na_2_O binary glass systems has been reported by Parsons et al.^11^ and Shih et al.,^12^ where the glass dissolution rate was found to significantly reduce with increasing amount of modifying oxides. Generally, trivalent cation oxides such as iron (Fe^3+^), titanium (Ti^3+^) and boron (B^3+^) are found to show greater influence on the solubility of phosphate glasses as compared to the divalent (Ca^2+^, Mg^2+^) and monovalent cation oxides.^13–17^

In recent years, there has been a growing interest in using borophosphate glasses as potential biomaterials, particularly due to the significant effect of B_2_O_3_ addition on the fibre-drawing process and also on the mechanical properties of the fibres.^1^ This ease of fibre formation and higher mechanical properties was attributed to the extension of phosphate chain length and increased crosslinking.^18^ Moreover, addition of B_2_O_3_ has also proven to improve the thermal and dissolution properties of PBGs. However, addition of B_2_O_3_ to PBGs did not show any favourable effect on the cell culture behaviour.^2^ It has already been proven that the addition of Fe_2_O_3_ to PBGs significantly improve the cytocompatibility of these glasses. However, the Fe_2_O_3_ does not impart any favourable effect on the fibre-drawing process. Therefore, the initial aim of this study was to evaluate the combined effect of B_2_O_3_ and Fe_2_O_3_ addition on the dissolution rate and dissolution mode of PBGs and fibres. The final aim was to relate the dissolution behaviour of B_2_O_3_ and Fe_2_O_3_ containing glasses with the cytocompatibility behaviour in order to evaluate the viability of these glasses as potential biomaterials. The phosphate content was fixed to 45 mol%. The effect of B_2_O_3_ and Fe_2_O_3_ addition on the thermal properties and density of PBGs was also evaluated.

## Materials and methodology

### Materials

Table 1 lists the precursors used in this study for making glass. All the precursors were purchased from Sigma–Aldrich, UK.

**Table 1.** Name and chemical formula of the precursors added during glass manufacture.

Name of the oxide required	Name and chemical formula of the precursor added
P_2_O_5_	Phosphorous pentoxide (P_2_O_5_)
CaO	Calcium hydrogen phosphate (CaHPO_4_)
Na_2_O	Sodium dihydrogen phosphate (NaH_2_PO_4_)
MgO	Magnesium hydrogen phosphate trihydrate (MgHPO_4_.3H_2_O)
B_2_O_3_	Boron oxide (B_2_O_3_)
Fe_2_O_3_	Iron (ΙΙΙ)-phosphate dehydrate (FePO_4_.2H_2_O)

Dulbecco’s Modified Eagle Medium (DMEM) consisting of DMEM (Gibco Invitrogen, UK) and 0.85 mM of ascorbic acid (Sigma–Aldrich) was used for cell culture studies. The supplementary components contained in DMEM are listed in Table 2.

**Table 2.** Name and concentration of the supplementary components present in Modified Eagle Medium (DMEM) for cell culture studies.

Supplementary component	Concentration in %
Foetal calf serum (FCS)	10
HEPES buffer	2
Penicillin/streptomycin	2
Glutamine	1
Non-essential amino acids	1

### Glass preparation

Melt-quenching process was used for the preparation of glass, where appropriate amount of precursors were mixed properly in a 200-mL volume Pt/5% Au crucible (Birmingham Metal Company, UK). The crucible containing the precursors was then moved to a furnace preheated to 350°C for half an hour for the removal of H_2_O, followed by melting in another furnace at 1150°C for 1.5 h. Finally, the molten glass was poured onto a steel plate and left to cool. As seen from Table 1, for all glass formulations, P_2_O_5_, CaO and MgO content were fixed to 45, 16 and 24 mol%, respectively, while the Fe_2_O_3_ content varied between 3 and 5 mol%. Two glass formulations contained 5 mol% B_2_O_3_ (coded as P45B5Fe3 (with 3 mol% Fe_2_O_3_) and P45B5F5 (with 5 mol% Fe_2_O_3_)), while other two did not contain any B_2_O_3_ (coded as P45Fe3 (with 3 mol% Fe_2_O_3_) and P45Fe5 (with 5 mol% Fe_2_O_3_)).

#### Thermal analysis

The thermal analysis of the glasses was conducted using a differential scanning calorimeter (TA Instruments SDT Q600, UK). The glass transition temperature (T_g_), onset of crystallisation (T_ons_), peak crystallisation (T_c_), melting (T_m_) and liquidus (T_L_) temperatures of the glasses were identified from individual thermal scans. A rotating ball mill was used to ground the glasses into fine powder for the analysis. The powdered glass samples were heated from room temperature to 1100°C a rate of 20°C per minute under flowing argon gas. Each composition was repeated three times.

#### Thermo-mechanical analysis

In order to determine the thermal expansion coefficient (α) of the glasses, a 9-mm graphite mould was preheated to 450°C and the molten glass was transferred into it. The glass was kept at 450°C for 1 h, and the temperature of the furnace was allowed to cool down to room temperature. Afterwards, the glass rods were preheated again to a temperature of 10°C above the glass transition temperature of individual glass composition at a rate of 5°C per minute and then cooled down to room temperature at a rate of 20°C per minute to remove the residual stresses from the glass rods. The glass rods were then cut using a low-speed saw into 7-mm-thick discs. Ethanol was used as lubricating oil for the cutting procedure. The 9 mm × 7 mm glass samples were heated at a rate of 5°C per minute with an applied load of 50 mN using a thermo mechanical analyser (TMA Q400, UK). The measured thermal expansion coefficient (α) was taken as an average between 50°C and 250°C. The whole experiment was conducted in triplicates.

#### Density measurement

Micromeritics AccuPyc 1330 helium pycnometer (Norcross, GA, USA) was used to identify the effect of composition on the density of the glasses which was calibrated using a standard calibration ball (3.18551 cm^3^) with errors of ±0.05%. Bulk glass samples were used for the density measurements.

#### Fibre production

Dedicated in-house facilities were used to produce fibres with ~20 µm diameter via a melt-drawn system where molten glass was exuded from a bushing and collected on a rotating drum in the form of fibre. The diameter of the fibres could be adjusted via adjusting the temperature of the glass melt or the speed of the collection drum.

#### Dissolution studies

For dissolution studies of the glass samples, the previously casted 9-mm diameter glass rods were cut into 5-mm discs in the same process as described in the ‘Thermo-mechanical analysis’ section. The height and diameter of each discs were measured, and the discs were dipped in 30 mL of phosphate buffer solution (PBS) contained in glass vials. These vials containing the glass discs in PBS solution were then placed into an incubator. The study was conducted at 37°C for 60 days. At various time points, the glass discs were dried off the PBS solution, and the area and mass of the glass discs were measured. At each time point, the previous PBS solution was drained out and fresh PBS solution was added. The dissolution rate of the glass rods was determined by plotting mass loss per area against time. The slope of this graph gave the dissolution rate in kg·m^−2^·s^−1^.

For the dissolution study, the fibres were cut into an average length of 50 mm and approximately 300 mg were placed vertically into the middle of individual glass vials, each containing 30 mL of PBS solution. The dissolution studies of the glass fibres were also conducted for 60 days. For the dissolution study of fibres, only P45Fe3 and P45B5Fe3 fibres were used as it was difficult to produce enough P45B5Fe5 fibres due to the high viscosity of the glass at the maximum attainable temperature of the fibre-drawing tower.

#### Cell culture

The cytocompatibility of the glass samples studied in this study was evaluated using MG63 cell lines (human osteosarcoma). The media and ascorbic acid were cultured in 75 cm^3^ flasks at 37°C in a humidified atmosphere with 5% CO_2_. For cell culture studies, 9-mm-diameter glass rods were cut into 2-mm-thick glass discs and then sterilised using dry heat (190°C) for 15 min followed by washing with sterilised PBS for three times. Concentration of the cells that were seeded onto the disc sample surfaces was 36,000 cells/cm^2^. The cell culture study was conducted for 7 days, and during this study period, the glass discs seeded with the cells were incubated at 37°C in a humidified atmosphere with 5 vol% CO_2_. The metabolic activity of the cells and the morphology of the cells cultured on the surface of the glass discs were conducted in the same way described in a previous publication.

## Results

### Thermal analysis

**Figure 1.**
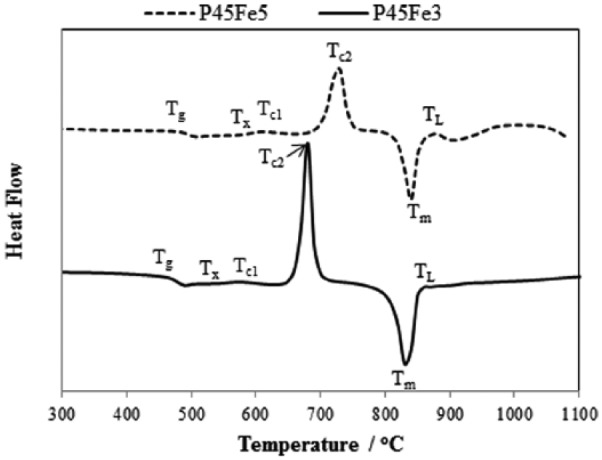
Thermal (DSC) scans for the P45Fe3 and P45Fe5 glasses.

**Figure 2.**
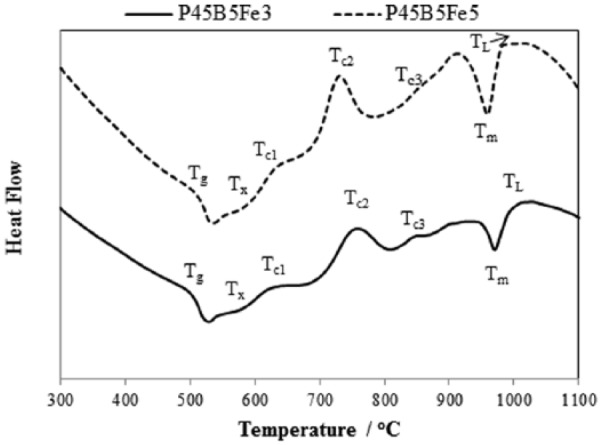
Thermal (DSC) scans for the P45B5Fe3 and P45B5Fe5 glasses.

Thermal scans of glasses investigated in this study are shown in Figure 1 (P45Fe3 and P45Fe5) and Figure 2 (P45B5Fe3 and P45B5Fe5). The corresponding glass transition temperature (T_g_), onset of crystallisation (T_c,ons_), crystallisation peak (T_c_), melting peak (T_m_) and liquidus (T_L_) temperature of the glasses have been reported in Table 3. As seen from Figure 1, T_g_ increased by ~32°C with increasing Fe_2_O_3_ content from 3 to 5 mol% for non-B_2_O_3_ containing glasses (P45Fe3 and P45Fe5). However, T_g_ only increased by ~11°C for 5 mol% B_2_O_3_ containing glasses as the Fe_2_O_3_ content increased from 3 to 5 mol% (P45B5Fe3 and P45B5Fe5). Addition of 5 mol% B_2_O_3_ to the 3 and 5 mol% Fe_2_O_3_ containing glasses caused T_g_ to increase by ~30°C. Similar to glass transition temperature, the onset of crystallisation also increased with B_2_O_3_ addition in each glass system. Non-B_2_O_3_ exhibited two crystallisation peaks (labelled as T_c1_ and T_c2_), while the crystalline peaks were observed for 5 mol% B_2_O_3_ containing glasses (labelled as T_c1_, T_c2_ and T_c3_). Moreover, P45Fe3 and P45Fe5 glasses exhibited sharper second crystalline peaks as compared to P45B5Fe3 and P45B5Fe5 glasses. One melting peak was observed for all the glass samples investigated and the melting temperature also shifted to higher temperature range with increasing B_2_O_3_ and Fe_2_O_3_.

**Table 3.** Thermal characteristics (T_x_, T_c_, T_m_ and T_L_) for P45Fe3, P45Fe5, P45B5Fe3 and P45B5Fe5 glasses.

Glass batches (mol%)	T_g_ (°C)	T_c,ons_ (°C)	T_c_ (°C)	T_m_ (°C)	T_L_ (°C)
P45Fe3	470 ± 1.0	542 ± 0.5	578.3 ± 0.7 681.6 ± 0.4	834.8 ± 0.3	851.3 ± 3.0
P45Fe5	485 ± 1.0	553 ± 1.0	604.6 ± 1.5 722.6 ± 0.3	838.6 ± 0.1	859.0 ± 0.1
P45B5Fe3	502 ± 1.0	580 ± 1.0	623.4 ± 0.8 755.5 ± 0.8 845.0 ± 0.8	974.2 ± 0.8	1010.0 ± 0.3
P45B5Fe5	513 ± 2.0	593 ± 1.0	631.0 ± 2.8 735.7 ± 2.7 857.0 ± 0.8	967.0 ± 1	1024.0 ± 0.3

### Thermal expansion

**Figure 3.**
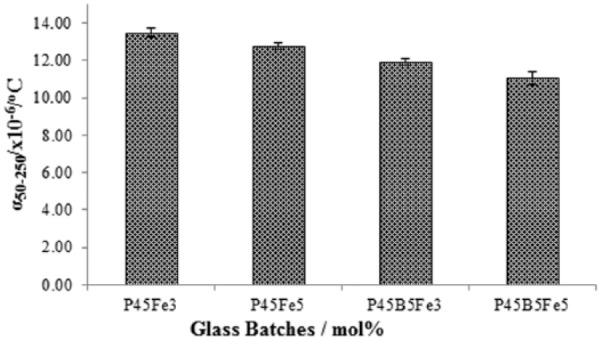
Thermal expansion coefficient (α) of P45Fe3, P45fe5, P45B5Fe3 and P45B5Fe5 glasses. Error bars represent the standard deviation where n = 3.

The glasses exhibited a decreasing trend in the thermal expansion coefficient values (denoted as α_50–250_) with increasing Fe_2_O_3_ and B_2_O_3_ content as shown in Figure 3. The thermal expansion coefficient values decrease from 13.48 × 10^−6^ °C to 12.76 × 10^−6^ °C as the Fe_2_O_3_ content increased from 3 to 5 mol%. A further decrease in thermal expansion coefficient from 11.92 × 10^−6^ °C to 11.03 × 10^−6^ °C was observed as 5 mol% B_2_O_3_ was added to the P45Fe3 and P45Fe5 glass systems, respectively.

### Density

**Figure 4.**
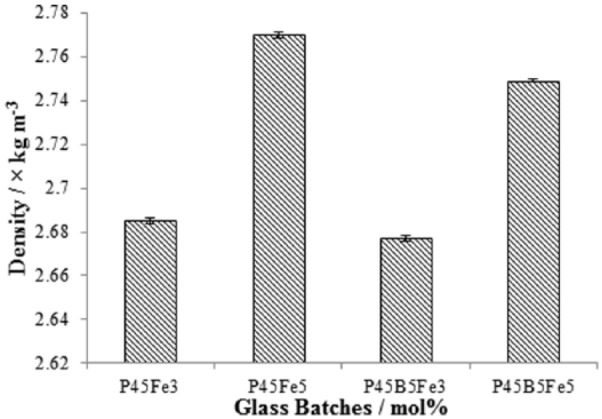
Density (ρ) of P45Fe3, P45fe5, P45B5Fe3 and P45B5Fe5 glasses. Error bars represent the standard deviation where n = 3.

Figure 4 shows the effect of Fe_2_O_3_ and B_2_O_3_ on the density of the glasses. The density was seen to increase with increasing Fe_2_O_3_ (3 to 5 mol%). The density of P45Fe3 was 2.68 × 10^3^ kg. m^−3^, whilst the density increased to 2.77 × 10^3^ kg. m−3 for P45Fe5 glasses. However, addition of B_2_O_3_ to glass systems was seen to decrease the density. The density of the P45Fe3 and P45Fe5 glass systems decreased to 2.67 × 10^3^ kg. m^−3^ and 2.74 × 10^3^ kg. m^−3^, respectively as 5 mol% of B_2_O_3_ was added to the glass systems.

### Dissolution studies

**Figure 5.**
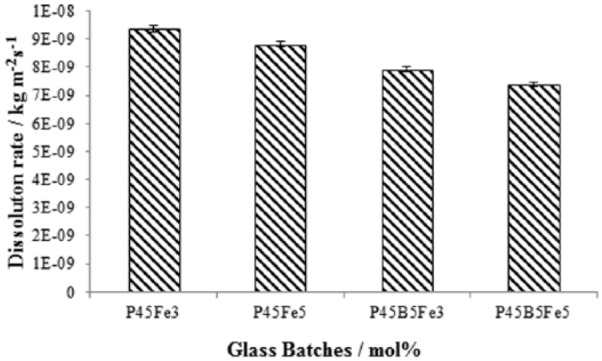
Dissolution rates obtained for P45Fe3, P45Fe5, P45B5Fe3 and P45B5Fe5 glasses. Error bars represent the standard deviation where n = 3.

**Figure 6.**
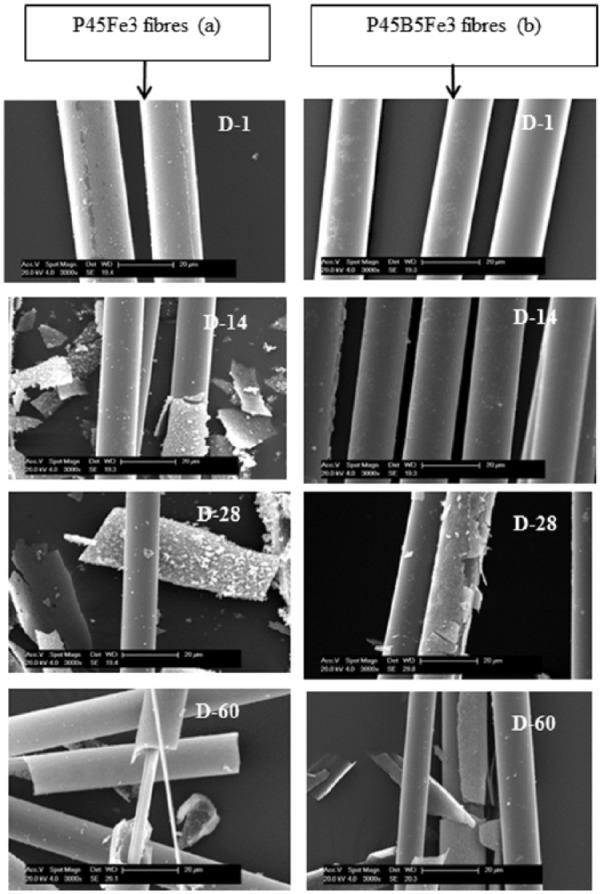
SEM images of (a) P45Fe3 non-annealed and (b) P45B5Fe3 non-annealed degraded fibres in PBS at 37°C after 1, 14, 28 and 60 days of solution degradation.

Figure 5 represents the dissolution rates obtained for the glasses investigated. A significant reduction in the dissolution rate was observed as Na_2_O was replaced with B_2_O_3_. A reduction in the dissolution rate was also observed with increasing Fe_2_O_3_ content from 3 to 5 mol%. The average dissolution rate of P45Fe3 and P45Fe5 glasses was 9.38 × 10^−9^ and 8.82 × 10^−9^ kg·m^−2^·s^−1^, respectively, after 60 days of immersion in PBS at 37°C. However, the dissolution rate of P45B5Fe3 and P45B5Fe5 glasses was 7.92 × 10^−9^ and 7.4 × 10^−9^ kg·m^−2^·s^−1^, respectively, which is significantly lower than the dissolution rate of P45Fe3 and P45Fe5 glasses as shown in Figure 5. Selected SEM images of the degraded P45Fe3 and P45B5Fe3 fibres are shown in Figure 6. As seen from the figure, both fibre types dissolved via the peeling of the outer layer. However, the dissolution mode of the fibres containing both B_2_O_3_ and Fe_2_O_3_ was less drastic as compared to the only Fe_2_O_3_ containing fibres.

### Cell culture

**Figure 7.**
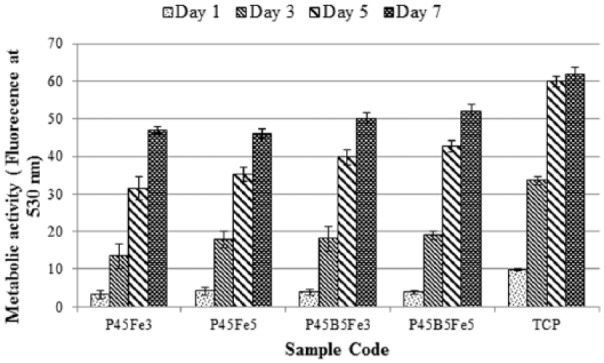
Metabolic activity of MG63 cells, as measured by the Alamar Blue assay, cultured on PBGs. The time points are Day 1, 3, 5 and 7. Error bars represent the standard deviation where n = 3.

The Alamar Blue assay was used to determine the effect of B_2_O_3_ and Fe_2_O_3_ addition on the metabolic activity of osteoblast-like cells (MG63). The cells were cultured for 7 days as shown in Figure 7. The time points of the cell culture were Day 1, 3, 5 and 7. For all glass samples investigated, the metabolic activity was seen to increase throughout the entire cell culture period. The polystyrene (TCP) control demonstrated an elevated metabolic activity compared to the all other glass samples investigated. At Day 1, there was no significant difference (p > 0.05) in the metabolic activity among the glass samples. At Day 5 and day 7, no significant difference (p > 0.05) in the metabolic activity among P45Fe3 and P45Fe5 was observed. However, the metabolic activity of P45B5Fe3 and P45B5Fe5 glass samples was significantly higher than the P45Fe3 and P45Fe5 glass samples at those two time points.

**Figure 8.**
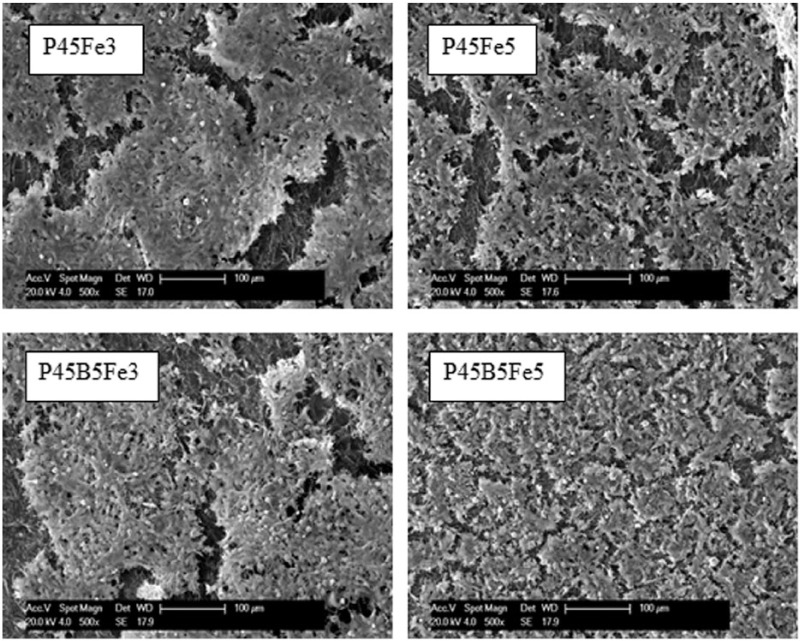
SEM images of MG63 cells cultured on P45Fe3, P45Fe5, P45B5Fe3 and P45B5Fe5 glass samples after 7 days of culture. Micrometer scale bar = 100 µm.

The SEM micrographs of MG63 cells cultured of P45Fe3, P45Fe5, P45B5Fe3 and P45B5Fe5 glass samples after 7 days of cell culture are presented in Figure 8. As seen from the figure, a dense multi-layered cell matrix was observed on all glass samples investigated after 7 days of cell culture.

## Discussion

The glass transition temperature (T_g_) increased by ~15°C as the Fe_2_O_3_ content increased from 3 to 5 mol% in both the glass systems, one with 5 mol% B_2_O_3_ and one without any B_2_O_3_. Addition of network modifiers to these glasses can result in network strengthening of the glass which could eventually lead to an increase in thermal properties and durability.^19^ The replacement of P-O-P bonds with P-O-M bonds (where, M = modifier cation) can result in increased glass transition temperature. Moreover, increasing amount of modifier oxides to the glass structure could potentially increase the cross-link density between the phosphate chains.^20^ The increase in T_g_ is also strongly influenced by thee increasing field strength of the interstitial cations due to increased cross-linking.^21^ Thus, the increased T_g_ with increasing Fe_2_O_3_ as observed in this study could be attributed to the increased cross-link density between the phosphate chains due to the formation of P-O-Fe bonds.^22^ Tan et al.^3^ studied the effect of 5, 8 and 11 mol% Fe_2_O_3_ addition on the thermal properties of MgO and CaO containing borophosphate glasses and reported a ~5°C increase in the glass transition temperature with every 3 mol% increase in the Fe_2_O_3_ content. Day et al.^23^ suggested that the presence of sharper crystalline peak is an indication of lower resistance of the glass towards crystallisation as compared to the one with broader crystalline peak. As observed from Figures 2 and 3, glasses with lower Fe_2_O_3_ content (3 mol%) showed sharper crystalline peak. Therefore, it could be concluded that the increased amount of Fe_2_O_3_ did increase the resistance of the glasses towards crystallisation.

The T_g_ values increased by ~30°C with 5 mol% addition of B_2_O_3_ to the 3 mol% Fe_2_O_3_ (P45Fe3) and 5 mol% Fe_2_O_3_ (P45Fe5) containing glasses. A similar effect on increasing B_2_O_3_ on T_g_ was observed in a previous publication; the T_g_ values increased by 60°C and 120°C with 10 mol% B_2_O_3_ addition to the glass systems with P_2_O_5_ content fixed to 45 and 50 mol%, respectively.^2^ The T_g_ value of glasses containing 5 mol% B_2_O_3_ with P_2_O_5_ content fixed to 45 mol% was reported to be ~473°C.^2^ However, the T_g_ value of P45B5Fe3 and P45B5Fe5 observed in this study was 502°C and 513°C, respectively. Therefore, the T_g_ of glasses containing both B_2_O_3_ and Fe_2_O_3_ was higher than the glasses with B_2_O_3_ or Fe_2_O_3_ alone. This increase in T_g_ was attributed to the higher field strength of B^3+^ as compared to the Na^+^.^2^ Generally, the presence of several crystalline peaks indicates the presence of several crystalline phases within the glass system.^15^ As discussed above, the presence of broader crystalline peaks could be concluded as more resistance of the glass structure towards crystallisation. Therefore, the addition of B_2_O_3_ can potentially increase the resistance of the iron phosphate glasses towards crystallisation which can positively affect the biocompatibility of these glasses.

The thermal expansion coefficient (α) is a very good indicative of how the glass would behave under different thermal shock.^24^ The α_50–250_ values showed a decreasing trend with increasing Fe_2_O_3_ content (see Figure 3). The α_50–250_ values decreased by 7% as the Fe_2_O_3_ content increased from 3 to 5 mol%. A further decrease in the α_50–250_ values of P45Fe3 and P45Fe5 glasses by ~15% was observed as 5 mol% B_2_O_3_ was added to the glasses. The α values of glasses are strongly influenced by the strength of glass network bonding, cross-linking and the interactions of the non-bridging oxygen with the cations.^1,20^ The network bonding and connectivity increases with the increasing cationic field strength, which is an indication of a cation’s effective force for attracting anions.^25^ The field strengths of both B^3+^ and Fe^3+^ cations are higher than Na^+^. Thus, because of the higher field strength, B^3+^ and Fe^3+^ would have a stronger ability to undergo coordination with other groups. Yu et al.^26^ investigated the effect of increasing Fe_2_O_3_ content from 14 to 43 mol% on the structure and properties of phosphate glasses and reported that the thermal expansion decreased with increasing Fe_2_O_3_. They suggested that reduction in thermal expansion coefficient with increasing Fe_2_O_3_ was an indication that the phosphorous–oxygen network became stronger due to the increased cross-linking between phosphate chains. Moreover, higher field strength–modifying oxides interacted strongly with the negatively charged phosphate anions and therefore hindered the mutual rotation and displacements of the anions which eventually decreased the thermal expansion. Thus, the increased cross-linking density with increasing Fe_2_O_3_ and B_2_O_3_ content decreased the basic structural vibrational movement of the glass, resulting in a decrease in the thermal expansion.

The density of the glasses is intensely affected by the field strength of the cations present in the glass structure. The presence of cations with higher field strength can form tighter bonding between phosphate chains as compared to the ones with lower field strength, which would eventually make the glass more compact and dense.^25^ In this study, B_2_O_3_ and Fe_2_O_3_ were added at the expense of Na_2_O. A monovalent cation (Na^+^) was replaced with trivalent cations (B^3+^ and Fe^3+^). Therefore, an increase in density was expected for both set of glasses. However, the density of the glasses only increased with increasing Fe_2_O_3_ (3 to 5 mol%; see Figure 4), and further addition of 5 mol% B_2_O_3_ decreased the density. The increase in density with increasing Fe_2_O_3_ could be associated with the higher field strength of iron as compared to sodium. Hasan et al.^27^ reported an increase in density with increasing iron content in the P_2_O_5_–CaO–MgO–Na_2_O–Fe_2_O_3_ glass system. They suggested that the density of the bulk glass was an important tool to measure the cross-link density and the packing structure of atoms, and the increased density with increasing Fe_2_O_3_ was due to the formation of Fe-O-P bonds and the structural changes associated with such change. Similar effect of increasing density with increasing Fe_2_O_3_ was also reported by Yu et al.^26^ The decrease in density of the glasses with B_2_O_3_ addition could be attributed to the increased chain length of the phosphate glass structure.^18^

The structure of pure PBGs without the presence of modifier oxides is composed of PO_4_ groups in chain/ring structures and many non-bridging oxygens.^1,13^ The -P-O-P- links present in the glass structure are known to hydrate easily and as such, P_2_O_5_ glasses, dissolve easily in aqueous media due to the fact that the 75% of the oxygen is bridging.^23^ Addition of modifying cations results in the partial depolymerisation of the glass network forming -P-O-M- (M/cation)-type links via the replacement of easily hydrated -P-O-P- bonds which consequently increase the chemical durability of the glasses. Addition of monovalent or divalent cations such as Na^+^ and Ca^2+^ can occupy ‘network modifying’ positions between the -P-O- chains, while multivalent cations such as B^+^ or Fe^3+^ may occupy ‘network forming’ positions by substituting a phosphorus ion in a -P-O-P- chain.^2^ Moreover, the addition of multivalent ions can create cross linking between the non-bridging oxygen of two phosphate chains which could also impart a positive effect on the durability of the glasses. As seen from Figure 5, the dissolution rate (by ~6%) was observed to decrease with increasing Fe_2_O_3_ (3 to 5 mol%) content. Fe can be present as Fe^2+^ and/or Fe^3+^ in the glass structure where Fe^3+^ can be present in both tetrahedral and octahedral coordination.^28^ Therefore, although Fe^2+^ acts as a network modifier, Fe^3+^ would act more like a network former, than a network modifier. Yu et al. studied the dissolution properties of sodium–iron phosphate glasses in water and also in saline at 90°C with iron content ranging from 14 to 43 mol% and with maximum Na_2_O content of 13 mol%. They reported that the increasing amount of Fe_2_O_3_ increased the durability of phosphate glasses both in water and in saline.^28^ Similar findings were also previously reported by Ahmed et al.^15^ and Hasan et al.^27^ Parsons et al.^11^ studied the effect of addition of different metal oxides on the dissolution rate of phosphate glasses and reported that the effect of different metal oxides goes in the order of Fe > Mg > Ca. Therefore, the decreased dissolution rate with increasing Fe_2_O_3_ was due to the replacement of P-O-P bonds with more hydration-resistant P-O-Fe bonds, and also due to the increased cross linking of the phosphate chains by iron ions.

A further reduction in the dissolution rate (~16%) was observed when 5 mol% B_2_O_3_ was added to the 3 mol% Fe_2_O_3_ (P45Fe3) and 5 mol% Fe_2_O_3_ (P45Fe5) containing glasses. As with Fe_2_O_3_ addition, the decrease in dissolution rate of the iron phosphate glasses with B_2_O_3_ addition was attributed to the replacement of P-O-P bonds with more hydration-resistant P-O-B bonds. Similar effect of B_2_O_3_ addition on the reduced dissolution rate of PBGs was reported in a previous publication.^2^ Kim et al.^29^ studied the dissolution property of borophosphate glasses in the system of xB_2_O_3_–(60–x)P_2_O_5_–40Na_2_O (x = 0, 10, 20, 30 and 40 mol%) and found that the dissolution rate of the glasses steeply decreased up to 30 mol% B_2_O_3_ addition. Ray et al.^30^ also suggested that the improvement in durability with the addition of boric oxide is related to the formation of durable BPO_4_ units. As mentioned in a previous publication, the dissolution rate of glasses with 5 mol% B_2_O_5_ and 45 mol% P_2_O_5_ with similar amount of CaO and MgO was 9.03 × 10^−9^ kg·m^−2^·s^−1^, whereas the dissolution rate of glasses containing both Fe_2_O_3_ and B_2_O_3_ is 7.92 × 10^−9^ kg·m^−2^·s^−1^ (for P45B5Fe3) and 7.40 × 10^−9^ k·gm^−2^·s^−1^ (P45B5Fe5), respectively. Therefore, addition of B_2_O_3_ together with Fe_2_O_3_ has more profound effect on the dissolution rate of the glasses than Fe_2_O_3_ or B_2_O_3_ alone.

Figure 8 shows the SEM images of P45Fe3 and P45B5Fe3 fibres degraded in PBS solution for 60 days. The P45Fe3 fibres went through more drastic degradation mode as compared to P45B5Fe3 glasses. The degradation of phosphate glass fibres usually takes place by peeling off the outer layer resulting in a decrease in the diameter of the fibres.^31^ In this study, we prepared ~20-µm-diameter fibres for the dissolution study. At the end of 60 days of dissolution study, the diameter of the P45B5Fe3 fibres reduced to ~15 µm, while for P45Fe3 fibres, the diameter of the degraded fibres went down to as low as ~8 µm. Therefore, the fibres containing both B_2_O_3_ and Fe_2_O_3_ were more resistant to hydrolytic attack, which is also consistent with the degradation studies conducted with the glass.

The SEM images of the MG63 cells cultured on the surface of the glasses investigated showed the presence of similar multi-layered dense cell layers after 7 days of cell culture. There was no significant difference in the metabolic activity between 3 and 5 mol% Fe_2_O_3_ containing glasses. However, the glasses containing Fe_2_O_3_ and B_2_O_3_ showed higher metabolic activity as compared to glasses containing Fe_2_O_3_ only. The biocompatibility of PBGs is strongly affected by the degradation rate. It is difficult for the cells to attach and proliferate on an unstable surface which might result from a high degradation rate.^1^ Moreover, lower degradation rate also helps to maintain the pH of the cell culture media suitable for cellular activity.^32^ It has already been well established that the addition of Fe_2_O_3_ could potentially enhance the biocompatibility of PBGs due to its positive effect on the chemical durability of these glasses.^13,15,27^ In a previous study, it was also reported that the addition of up to 5 mol% B_2_O_3_ into 45 and 50 mol% P_2_O_5_ containing glasses showed favourable cellular response.^2^ Zhu et al.^33^ studied the effect of increasing B_2_O_3_ content from 12 to 20 mol% on the cell metabolic activity and proliferation of PBGs. They suggested that the metabolic activity of the glasses containing 15 and 20 mol% B_2_O_3_ was significantly lower than the glasses with 12 mol% B_2_O_3_ due to the higher dissolution rate of the glasses. Fu et al.^34^ suggested that the accepted concentration level of boron in B_2_O_3_ containing bioactive glasses should be equal to or below 0.65 mM in order for them to be used as potential biomaterials. Therefore, from the cell culture studies, it could be concluded that the boron ion released from the glasses in this study did not impart any negative effect on the cytocompatibility of the glasses, rather the higher chemical durability of glasses with Fe_2_O_3_ and B_2_O_3_ made them more suitable for different biomedical applications.

## Summary

In this article, Four PBG compositions were produced by replacing Na_2_O with B_2_O_3_ and/or Fe_2_O_3_ in the glass system P_2_O_5_–CaO–Na_2_O–MgO, and the P_2_O_5_ content was fixed at 45 mol%. It was not possible to determine the proportion of Fe^2+^or Fe^3+^ oxides in this study. T_g_, T_c_, T_L_ and T_m_ increased as Na_2_O was replaced with B_2_O_3_ and/or Fe_2_O_3._ The highest T_g_ (513°C) was observed for glasses with 5 mol% Fe_2_O_3_ and/or FeO and 5 mol% B_2_O_3_ (P45B5Fe5). However, the thermal expansion coefficient values, density and dissolution glasses containing both B_2_O_3_ and Fe_2_O_3_ were significantly lower than the only B_2_O_3_ or Fe_2_O_3_ containing glasses. The improved physical properties of the glasses investigated with the addition of B_2_O_3_ and Fe_2_O_3_ were attributed to the replacement of P-O-P bonds with P-O-B and P-O-Fe bonds. The in vitro cell culture studies revealed that both P45B5Fe3 and P45B5Fe5 glasses maintained and showed higher cell viability as compared to the P45Fe3 and P45Fe5 glasses. This was attributed to the slower dissolution rate of P45B5Fe3 and P45B5Fe5 glasses as compared to P45Fe3 and P45Fe5.
